# Nutrition Intervention Informed by Indirect Calorimetry Compared to Predictive Equations to Achieve Weight Goals in Geriatric Rehabilitation Inpatients: The NEED Study

**DOI:** 10.1007/s12603-023-1970-5

**Published:** 2023-09-23

**Authors:** J. Hettiarachchi, K. Fetterplace, Andrea B. Maier, E.M. Reijnierse

**Affiliations:** 1 Department of Medicine and Aged Care, @AgeMelbourne, The Royal Melbourne Hospital, The University of Melbourne, Parkville, VIC, Australia; 2 Department of Allied Health (Clinical Nutrition), The Royal Melbourne Hospital, Melbourne, VIC, Australia; 3 Department of Critical Care, Melbourne Medical School, The University of Melbourne, Melbourne, VIC, Australia; 4 Department of Human Movement Sciences, @AgeAmsterdam, Faculty of Behavioral and Movement Sciences, Vrije Universiteit Amsterdam, Amsterdam Movement Sciences, Van der Boechorststraat 7, 1081 BT, Amsterdam, The Netherlands; 5 Healthy Longevity Program, Yong Loo Lin School of Medicine, National University of Singapore, Singapore, Singapore; 6 Centre for Healthy Longevity, @AgeSingapore, National University Health System, Singapore, Singapore; 7 Centre of Expertise Urban Vitality, Faculty of Sports and Nutrition, Amsterdam University of Applied Sciences, Amsterdam, The Netherlands

**Keywords:** Indirect calorimetry, body weight, muscle mass, older adults, inpatients, energy targets

## Abstract

**Objectives:**

To assess if nutritional interventions informed by indirect calorimetry (IC), compared to predictive equations, show greater improvements in achieving weight goals, muscle mass, strength, physical and functional performance.

**Design:**

Quasi-experimental study.

**Setting and Participants:**

Geriatric rehabilitation inpatients referred to dietitian.

**Intervention and Measurements:**

Patients were allocated based on admission ward to either the IC or equation (EQ) group. Measured resting metabolic rate (RMR) by IC was communicated to the treating dietitian for the IC group but concealed for the EQ group. Achieving weight goals was determined by comparing individualised weight goals with weight changes from inclusion to discharge (weight gain/loss: >2% change, maintenance: ≤2%). Muscle mass, strength, physical and functional performance were assessed at admission and discharge. Food intake was assessed twice over three-days at inclusion and before discharge using plate waste observation.

**Results:**

Fifty-three patients were included (IC n=22; EQ n=31; age: 84.3±8.4 years). The measured RMR was lower than the estimated RMR within both groups [mean difference IC −282 (95%CI −490;−203), EQ −273 (−381;−42) kcal/day)] and comparable between-groups (median IC 1271 [interquartile range 1111;1446] versus EQ 1302 [1135;1397] kcal/day, p=0.800). Energy targets in the IC group were lower than the EQ group [mean difference −317 (95%CI −479;−155) kcal/day]. There were no between-group differences in energy intake, achieving weight goals, changes in muscle mass, strength, physical and functional performance.

**Conclusions:**

In geriatric rehabilitation inpatients, nutritional interventions informed by IC compared to predictive equations showed no greater improvement in achieving weight goals, muscle mass, strength, physical and functional performance. IC facilitates more accurate determination of energy targets in this population. However, evidence for the potential benefits of its use in nutrition interventions was limited by a lack of agreement between patients' energy intake and energy targets.

## Introduction

**M**alnutrition is highly prevalent in geriatric rehabilitation patients (1) and is associated with negative outcomes including poor physical and functional performance (2), poor quality of life, institutionalisation and higher mortality (3). Nutritional interventions have shown to improve nutritional status and functional outcomes in geriatric rehabilitation patients (4). Providing adequate energy to meet individualised nutritional requirements and improve nutritional status, consequently to maintain or improve function is a priority aim in clinical nutrition (5), particularly in geriatrics and rehabilitation (6).

Unintentional weight loss is a characteristic of poor nutritional status and is associated with higher morbidity and mortality in older adults (7). Therefore, weight management is an important goal of nutritional interventions that requires provision of energy to meet energy requirements and to achieve individualised weight goals (8). Daily energy requirements are predominantly (60 to 70%) determined by resting metabolic rate (RMR), the energy required to maintain body functions at rest (9). In clinical practice, predictive equations are often used to estimate RMR. However, predictive equations were originally derived from healthy adult populations and do not account for body composition, disease(s), and age-related metabolic changes in older adults (10). Discrepancies between estimated versus measured RMR by indirect calorimetry were found in older adults hospitalised with malnutrition and critical illnesses. The estimated RMR varied by more than 10% of the measured RMR in more than half of the patients leading to over or underestimation of the actual energy requirements (12, 13). Indirect calorimetry is the gold standard method to measure RMR providing an accurate estimation of total energy requirements (11). However, it is unknown if utilising indirect calorimetry in determining energy requirements can lead to greater improvements in achieving weight goals and clinical outcomes compared to predictive equations in geriatric patients.

This study aimed to assess if nutritional interventions informed by indirect calorimetry measurements, compared to predictive equations, can lead to a higher proportion of patients achieving their weight goals (primary outcome) and greater improvements in muscle mass, muscle strength, physical and functional performance (secondary outcomes) in geriatric rehabilitation inpatients.

## Materials and Methods

### Study design

Nutrition, Energy Expenditure, and Demands (NEED) is a sub-study within the wider REStORing health of acutely unwell adulTs (RESORT) cohort of geriatric rehabilitation inpatients admitted to a university-affiliated hospital (Melbourne, Victoria, Australia). All patients admitted between 15th October 2017 and 18th March 2020 were included in RESORT. Patients were excluded if they were receiving palliative care at admission, had no capacity to provide informed consent and/or had no nominated proxy to consent on their behalf. All patients were assessed by physicians, nurses, physiotherapists, occupational therapists and dietitians (if referred) using the Comprehensive Geriatric Assessment (CGA) within 48 hours of admission and 48 hours before discharge as a part of standard care. The study was approved by the Melbourne Health Human Research Ethics Committee (HREC/17/MH/103) and followed national and international ethical guidelines according to the Helsinki Declaration (14).

NEED is a quasi-experimental study with a cluster crossover design and included patients referred to the dietitian and consented to the RESORT study between 28th May 2019 and 19th March 2020. Patients were screened for eligibility for NEED and included within 72 hours of dietitian referral. Patients were excluded in case of RMR measurement contraindications (severe dementia, delirium, contact isolation, using breathing apparatus, severe claustrophobia), if included in another study where patients perform resistant exercises additional to routine care or treating physician not supporting inclusion. Informed consent was obtained from the eligible patients or the nominated proxy to be included in NEED. If an eligible patient developed a contraindication after inclusion in NEED and the RMR measurement could not be performed within 72 hours of dietitian referral, such patients were considered dropped out. A total sample of 60 patients with 30 in each group was targeted which is considered to be adequate for a feasibility study identifying the potential to progress in to larger definitive studies (15). Patients were assigned to the indirect calorimetry (IC) group or the equation (EQ) group according to the admission ward. Out of the four geriatric rehabilitation wards in the hospital, two wards were initially allocated as the IC group and the other two wards as the EQ group. After recruiting a minimum of 15 patients in each group, the wards crossed over with the IC and EQ group. Recruitment was ceased on 19^th^ March 2020 before reaching the anticipated sample size due to the COVID-19 pandemic. Figure 1a shows a schematic of the NEED study.

**Figure 1a:**
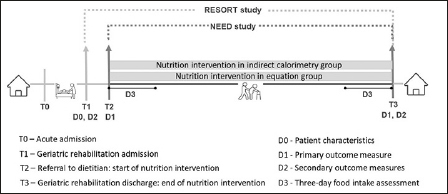
Schematic of NEED

T indicates time points, D indicates data collection

### Patient characteristics

Patient demographics including age and sex were obtained from medical records. Information on the living situation (living alone) was obtained from surveys completed by patients and or caregivers. The length of hospital stay in the geriatric rehabilitation ward and the primary reason for hospitalisation were extracted from medical records. The reasons for hospitalisation were categorised into musculoskeletal, neurological, infection, cardiorespiratory related conditions or others. Frailty status and comorbidity were assessed by physicians using the clinical frailty scale (9-point scale with 1 indicating very fit to 9 indicating terminally ill) (16) and Charlson comorbidity index (a higher score indicating higher comorbidity) (17). Cognitive impairment was defined based on physicians' assessment if: dementia or mild cognitive impairment/minor neurocognitive disorder reported as a diagnosis in medical records or indicated on the CCI; standardised Mini-Mental State Examination (sMMSE) (18) score of <24 points, a Montreal Cognitive Assessment (MoCA) (19) score <26 points, or a Rowland Universal Dementia Assessment Scale (RUDAS) (20) score <23 points. Delirium was identified by physicians as a delirium diagnosis or risk of delirium according to the short Confusion Assessment Method (21). Anthropometry data were collected by nurses including body weight to the nearest 0.1 kg using a calibrated weighing scale, weighing chair or hoist, and standing height or knee height to the nearest 0.1 m dependent on the patients' ability to stand. The knee height was converted to standing height using the Chumlea equation for Caucasians (22). Body mass index (BMI) was calculated as weight divided by height squared (kg/m^2^). Patients were screened for the risk of malnutrition by nurses using the malnutrition screening tool (MST); a score of ≥2 points was considered as at risk of malnutrition (23).

### Nutritional assessment

The nutritional status was assessed by the researchers using the Mini Nutritional Assessment (MNA) long-form. Patients with a MNA score <17.0 points, 17.0–23.5 points, and >23.5 points out of 30.0 points were identified as malnourished, at risk of malnutrition, and well-nourished respectively (24).

The RMR of all patients included in NEED was measured using indirect calorimetry with a canopy system (Fitmate GS, COSMED, Rome, Italy) by trained researchers within 72 hours of dietitian referral. The Fitmate GS measures the volume of oxygen consumed (VO2) and estimates the volume of carbon dioxide produced using a fixed respiratory quotient of 0.85 based on the abbreviated Weir equation (25) to provide the RMR value. The Fitmate GS has shown good relative agreement in ambulatory and hospitalised patients with the reference standard device, the DELTATRAC II metabolic monitor (26). The equipment was calibrated daily according to the manufacturer's guidelines. The measurements took place 1.5–3 hours post breakfast and before patients performed any strenuous activity or undertook physiotherapy. Information on patients' last meal and drink, if the patient smoked before the measurement and the use of thyroxine were recorded as these factors could influence the RMR. Patients were instructed to lie still in supine position on the hospital bed with limited movement and talking while breathing normally. The canopy hood was placed over the patient's head and the measurement continued for 30 minutes. If patients requested to cease the measurement before 30 minutes but completed at least 20 minutes and reached the steady state, the measurement was included in the analyses. Patients were excluded if the steady state was not reached, or measurement duration was <20 minutes. A steady state was identified when the coefficient of variation in VO2 was <10%. A trained researcher monitored the patient throughout the measurement. The first 5 minutes of the measurement data were discarded as the patient was adapting to breathing under the canopy hood. The average of data between 5 minutes and the end of measurement was used as the RMR and the VO2 (27).

In the IC group, measured RMR of the patient was communicated to the dietitians whereas it was concealed from the dietitians in the EQ group. The intervention energy targets for the patients in the IC group were determined using the measured RMR and the individual physical activity factor as determined by the treating dietitian. In the EQ group, energy targets were determined by dietitians using a suitable equation of their clinical judgement (predominantly the Schofield equation (28) unless indicated otherwise), applying the stress factor and physical activity factor as appropriate for the individual patient, which was the routine clinical practice. Individualised weight goals (gain/maintenance/loss) were set by the dietitian and the nutrition intervention energy and protein targets were adjusted accordingly. All dietitians followed local clinical practice guidelines based on current international guidelines for the delivery of nutrition interventions. Therapeutic diet provision, oral nutrition supplements and nutrition education were used by dietitians as indicated to meet individualised energy and protein targets.

The patients' food intake was assessed by the researcher using plate waste observation at two time points, the first over three consecutive days starting from the day of inclusion and the second over three days before discharge. The food consumed at all main meals (breakfast, lunch, and dinner) was recorded by taking photographs of the patients' meal trays pre and post mealtimes. The food intake was assessed based on if the patient consumed a quarter, half, three-quarter, or all, of each item severed on the meal tray. These proportions were entered into the mobile intake data application of the Room Service program available on the CBORD electronic menu management system (Tray Monitor, CBORD® Group Inc.) to calculate patients' energy and nutrient intake (29). Information on any food item consumed additionally to the hospital meals was obtained by interview with the patients or the carers. The snacks consumed during mid-morning and afternoon were recorded. The nutrient composition of the additional food items and snacks was calculated using the Foodworks 9 Professional software. The energy and protein content in each served meal was calculated using the nutrient composition of the menu items and snack items. The three-day average energy and protein intake and the amount of energy and protein served were calculated for each patient at each time point.

### Outcome measures

Patients were weighed within 48 hours before the inclusion and before discharge. The absolute weight change was obtained by deducting the weight at the inclusion from the weight at discharge. The primary outcome was achieving individualised weight goals and was confirmed by comparing the weight change to the weight goal set by the treating dietitian. Weight gain or weight loss was defined as the weight change >2% of the weight at inclusion and weight maintenance as ≤2% of the weight at inclusion (30).

Secondary outcomes, including the change in muscle mass, muscle strength, physical and functional performance, were derived from the CGA at admission and discharge from geriatric rehabilitation wards. Muscle mass was measured by nurses using a direct segmental multi-frequency bioelectrical impedance analysis (BIA) in supine position (DSM-BIA, In-Body S10, Biospace Co., Ltd, Seoul, South Korea) (31). Muscle mass was expressed as appendicular lean mass (ALM) (kg) and appendicular lean mass index (ALMI) (kg/m^2^) calculated as the ALM divided by height squared. Muscle strength was measured by the handgrip strength (kg) and was assessed by physiotherapists using a handheld dynamometer (Sammons Preston, Inc., Bolingbrook, IL, USA). Patients were encouraged to squeeze the dynamometer with their maximum strength in a seated position with the elbow bend at 90 degrees and without the arm touching the trunk. Three attempts were given for each hand alternating; the maximum strength by either right or left hand was used as the handgrip strength in the analysis (32). Physical performance was assessed by physiotherapists using the Short Physical Performance Battery (SPPB). The total score of the SPPB ranged from 0–12 with higher scores showing better physical performance. The chair stand test (CST) and gait speed tests were also separately analysed. For the CST, patients were asked to do five timed rises from a chair to a fully upright position without using arms consecutively as fast as possible. Scores were given as 0 if unable, 1, 2, 3, and 4 if time in seconds ≥16.70, 13.70 – 16.69, 11.20 – 13.69, and ≤11.19 respectively (33). Gait speed (m/s) was assessed as the fastest out of two attempts in a timed four-meter walk at usual pace. Functional performance was assessed by occupational therapists using the Katz index for activities of daily living (ADL) with a score ranging 0–6 (34) and the Lawton and Brody scale for instrumental activities of daily living (IADL) with a score ranging 0–8 (35), higher scores in both scales indicating better functional performance.

### Statistical analysis

Continuous variables with normal distributions are presented as means ± standard deviations (SD) and non-normal distributions as medians [interquartile ranges] [IQR]. Categorical variables are presented as frequencies (n) and percentages (%).

Between-group differences in patient characteristics and secondary outcome measures at admission were assessed using the Mann-Whitney U test for continuous variables and Chi-square tests for categorical variables. Mann-Whitney U tests were used to compare the between group differences in RMR, energy and protein target, served and intake at inclusion and at discharge. Between group differences were presented as the mean difference and 95% confidence intervals (CI). Within group differences in measured versus estimated RMR, energy target versus energy served, energy intake versus energy target and protein intake versus protein target at inclusion and at discharge were determined using the Wilcoxon rank-sum test and presented as mean difference (MD) with 95% CI.

Bland-Altman plots were generated to visualize the agreement between measured versus estimated RMR, energy targets versus energy served, energy intake versus energy target and protein intake versus protein target at inclusion and at discharge among patients at the individual level in each group (36). Proportional bias was determined by a statistically significant deviation of the slope of the regression line in the Bland-Altman plots for the difference against the average of RMR measured and estimated, energy target and energy served, energy intake and energy target and protein intake and protein target at inclusion and at discharge.

To compare the between group differences in the proportion of patients achieving weight goals (primary outcome) chi-square test was used. Within-group changes in secondary outcomes from admission to discharge were assessed using the Wilcoxon rank-sum test and presented as mean change with 95% CI. Between group difference in absolute weight change (inclusion to discharge) and change in secondary outcomes (admission to discharge) were determined using Mann-Whitney U tests and presented as the MD and 95% CI. All statistical analyses were performed using the Statistical Package for the Social Sciences version 25.0 (IBM SPSS Advanced Statistics 25.0, Armonk, NY, IBM Corp). A p-value of less than 0.05 was considered statistically significant.

## Results

### Patient characteristics

Of the 414 geriatric rehabilitation patients referred to dietitians, 358 were included in RESORT and screened for eligibility for NEED. Out of those patients, 66 were recruited and 53 completed the study. The main reasons for exclusion from NEED were severe dementia and/or delirium (n=57), the treating physician advised against inclusion (n=47), inclusion in another study (n=22), contact isolation (n=21) (Figure 1b). Thirteen patients dropped out: ten experiencing discomfort and/or claustrophobia during the RMR measurement, two showing signs of delirium as per treating physician's opinion and one feeling unwell on the day of measurement. Patient characteristics at admission are presented in Table 1. There were no between group differences in demographic characteristics, frailty, cognitive status, comorbidity and nutritional characteristics. In the EQ group, a higher number of patients had musculoskeletal conditions as the reason for hospitalisation and patients had lower gait speed and ADL score compared to the IC group at admission.

**Figure 1b:**
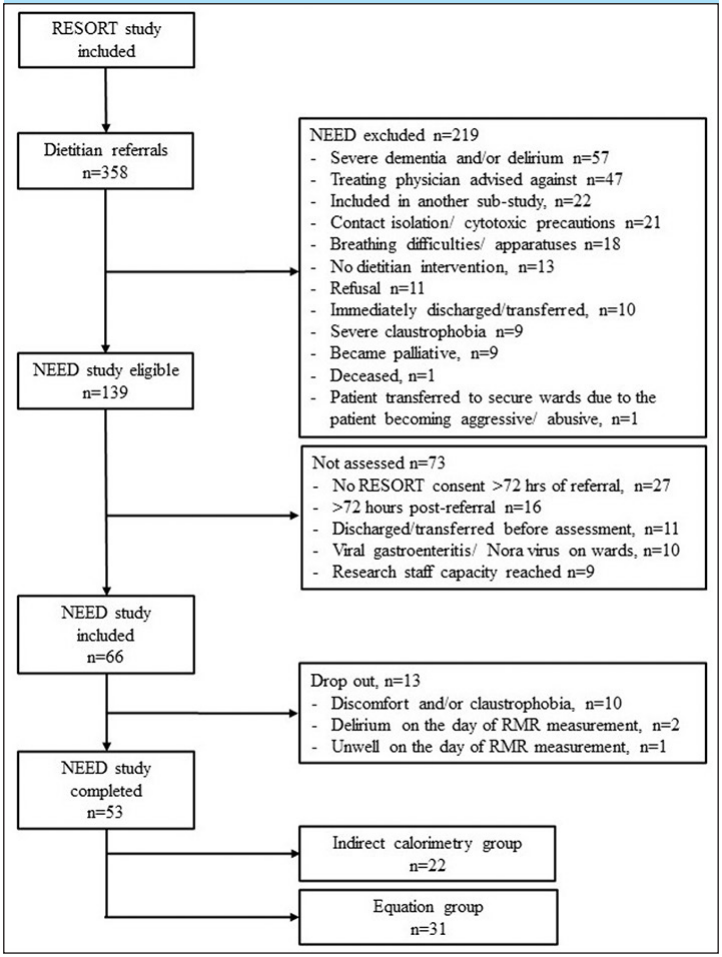
Patient screening and inclusion in NEED

RMR: Resting metabolic rate

**Table 1:** Patient characteristics of indirect calorimetry and equation groups (N = 53)

**Characteristic**	**Indirect calorimetry (n = 22)**		**Equation (n = 31)**		**p-value**
	**n**	**Value**	**n**	**Value**	
Demographics and health status at admission					
Age, years, mean (SD)	22	84.3 (6.6)	31	84.3 (9.6)	0.971
Female, n (%)	22	8 (36.4)	31	14 (45.1)	0.522
Living alone, n (%)	22	14 (63.6)	31	20 (64.5)	0.240
Length of stay, days	22	20 [18; 33]	31	35 [21; 50]	0.030
Clinical frailty scale, score	18	6 [5; 7]	30	6 [5; 7]	0.745
Cognitive impairment, n (%)	22	14 (63.6)	31	18 (58.1)	0.683
Charlson comorbidity index, score	22	2 [1; 4]	31	3 [1; 4]	0.661
Reason for hospitalisation, n (%)	22		31		0.026
Musculoskeletal, n (%)		7 (31.8)		21 (67.7)	
Neurological, n (%)		1 (4.5)		5 (16.1)	
Infection, n (%)		1 (4.5)		0 (0)	
Cardiorespiratory, n (%)		7 (31.8)		1 (3.2)	
Other^a^, n (%)		6 (27.3)		4 (12.9)	
Body mass index, kg/m^2^	22	23.1 [20.3; 25.3]	31	24.2 [21.5; 27.8]	0.406
Malnutrition Screening Tool, score	22	1 [0–2]	31	2 [0–2]	0.341
Mini Nutritional Assessment	22		31		0.219
Well-nourished, n (%)		0 (0)		2 (6.5)	
At risk of malnutrition, n (%)		14 (63.6)		23 (74.2)	
Malnutrition, n (%)		8 (36.4)		6 (19.3)	
Weight, kg	22	65.3 [55.9; 77.1]	31	69.5 [56.9; 79.4]	0.454
Secondary outcome measures					
ALM, kg	19	18.5 [15.0; 21.6]	20	21.0 [18.2; 24.0]	0.251
ALMI, kg/m^2^	19	6.5 [6.0; 8.0]	20	7.7 [6.7; 8.9]	0.071
Handgrip strength, kg	22	20.5 [15.0; 25.0]	27	17.0 [10.5; 22.0]	0.862
CST, score	22	0 [0–1]	28	0 [0 0]	0.167
Gait speed, m/s	22	0.46 [0.00; 0.64]	28	0.00 [0.00; 0.39]	0.034
SPPB, score	21	2 [0–5]	28	1 [0–5]	0.290
Katz-ADL, score	22	2 [1–3]	31	1 [1–2]	0.015
Lawton-IADL, score	22	1 [0–2]	31	1 [1–2]	0.087


ALM: Appendicular lean mass; ALMI: Appendicular lean mass index; CST: Chair stand test; SPPB: Short physical performance test; ADL: Activities of daily living; IADL: Instrumental ADL; Data are presented as median [IQR] unless indicated otherwise. IQR: interquartile range; SD: standard deviation. ^a^Other include gastrointestinal, urology, hematology, ophthalmology, vascular, psychiatry related conditions, cancer, and metabolic disorders

### Resting metabolic rate, energy target and energy served

Table 2 shows the nutrition intervention characteristics in the IC and EQ groups. There were no between group differences in the RMR measured by indirect calorimetry and the RMR estimated by equations. The measured RMR was significantly lower than the estimated RMR by equations within both groups at population level [MD (95%CI) IC: −282 (−490; −203) kcal/ day, p = 0.001, EQ: −273 (−381; −42) kcal/day, p = 0.001]. Bland-Altman plot showed wide limits of agreement (LOA) for the measured versus estimated RMR at the individual level (95% LOA −671 to 41 kcal/day) (Supplementary figure 1).

**Table 2:** Nutrition intervention characteristics in indirect calorimetry and equation groups

**Characteristic**	**Indirect calorimetry**		**Equation**		**Between-group differences**	
	**n**	**Value**	**n**	**Value**	**Mean difference [95% CI]**	**p-value**
Resting metabolic rate (RMR)						
RMR measured (kcal/day)	22	1271 [1111; 1446]	31	1302 [1135; 1397]	−20 [−147; 108]	0.800
RMR measured (kcal/kg of body weight/day)	22	19.8 [17.4; 20.7]	31	18.6 [18.0; 20.4]	−0.1 [−2.1; 1.8]	0.909
RMR estimated by equations ^a^ (kcal/day)	22	1587 [1462; 1803]	31	1603 [1419; 1687]	30 [−85; 146]	0.527
RMR measured – RMR estimated (kcal/day)	22	−282 [−490; −203]	31	−273 [−381; −42]	−50 [−152; 52]	0.787
Energy and protein targets and served						
Energy target (kcal/day)	22	1525 [1321; 1735]	31	1793 [1625; 1960]	−317 [−479; −155]	0.001
Energy served (kcal/day)	22	2044 [1750; 2425]	31	2080 [1884; 2535]	−4 [−284; 277]	0.665
Energy served – energy target (kcal/day)	22	446 [117; 775]	31	311 [116; 506]	57 [−274; 388]	0.986
Protein target (g/kg/day)	22	1.2 [1.2; 1.2]	31	1.2 [1.2; 1.2]	0 [−0.1; 0.1]	0.886
Protein served (g/kg/day)	22	1.4 [1.1; 2.0]	31	1.4 [1.1; 1.7]	0.1 [−0.2; 0.4]	0.773
Length of intervention ^b^, days	22	15 [9; 24]	31	24 [15; 34]	−5 [−14; 4]	0.064
Type of interventions used^c^						
Therapeutic diet provision, n (%)	22	22 (100.0)	31	30 (96.7)		0.574^d^
Oral nutrition supplementation, n (%)	22	11 (50.0)	31	19 (61.2)		
Energy and protein intake at inclusion						
Energy intake (kcal/day)	22	1482 [1110; 2108]	31	1610 [1213; 1940]	43 [−236; 322]	0.857
Energy difference ^e^ (kcal/day), mean [95% CI]	22	−97 [−314; 121]	31	−245 [−418; −73]	149 [−119; 417]	0.220
Protein intake (g/kg/day)	22	1.1 [0.8; 1.5]	31	1.0 [0.8; 1.3]	0.2 [−0.1; 0.4]	0.665
Protein difference ^e^ (g/kg/day), mean [95% CI]	22	−0.1 [−0.5; 0.3]	31	−0.2 [−0.3; 0.0]	0.1 [−0.1; 0.4]	0.504
Protein difference ^e^ (g/day), mean [95% CI]	22	−7.1 [−24.0; 9.7]	31	−13.4 [−24.0; −2.7]	6.3 [−12.1; 24.8]	0.367
Energy and protein intake at discharge						
Energy intake (kcal/day)	10	1598 [1207; 1722]	19	1393 [1122; 1824]	−60 [−439; 318]	0.740
Energy difference ^e^ (kcal/day), mean [95% CI]	10	−167 [−483; 149]	19	−148 [−400; 104]	291 [−25; 606]	0.077
Protein intake (g/kg/day)	10	1.1 [0.8; 1.6]	19	1.0 [0.8; 1.2]	0.2 [−0.1; 0.5]	0.350
Protein difference ^e^ (g/kg/day), mean [95% CI]	10	−0.1 [−0.3; 0.4]	19	−0.3 [−0.5; −0.1]	0.3 [−0.1; 0.6]	0.151
Protein difference ^e^ (g/day), mean [95% CI]	10	−9.0 [−41.0; 23.6]	19	−15.0 [−27.0; −3.0]	6.1 [−20.3; 32.5]	0.247


RMR: Resting metabolic rate. Values are given as median [IQR] unless otherwise stated. Bold p-values are statistically significant. ^a^Determined for individual patients using RMR by equation times the stress factor determined by the dietitian, ^b^Calculated as the number of days between the commencement of nutrition intervention by dietitian and discharge from the ward, ^c^Nutrition education was given to all patients/caretakers and an individual patient may have received multiple interventions, ^d^p-value obtained from the chi-square test, ^e^Difference is calculated as the intake minus the target.

The median physical activity factor determined by the dietitians was 1.15 [IQR: 1.15–1.20] for patients in both groups and the median stress factor was 1.20 [IQR:1.15–1.25] for patients in the EQ group. The energy target in the IC group was lower than the EQ group [MD (95%CI) −317 (−479; −155) kcal/day, p = 0.001] and the energy served was not different between groups. The energy served was significantly higher than the energy target within both groups at group level [MD (95% CI) IC: 446 (117 to 775) kcal/day, p = 0.001, EQ: 311 (116 to 506) kcal/day, p = 0.002]. Bland-Altman analysis showed a significant proportional bias between the energy served and energy targets at the individual level (β = 0.735, p = 0.019) (Supplementary figure 2).

### Energy and protein intake and targets

No between group differences were found in energy and protein intake both at inclusion and at discharge. Within the IC group, the energy intake was comparable to the energy targets at inclusion [MD (95% CI) −97 (−314; 121) kcal/day] and discharge [MD (95% CI) −167 (−483; 149) kcal/day]. In the EQ group, the energy intake was below the target at inclusion [MD (95% CI) −245 (−418; −73) kcal/day, p = 0.011], but not significantly different at discharge [MD (95% CI) −148 (−400; 104) kcal/day]. At the individual level, Bland-Altman analyses showed a wide LOA for energy intake versus energy target both at inclusion and discharge in the IC group and at discharge in the EQ group (IC: 95% LOA −1057 to 864 and − 1032 to 698 kcal/day at inclusion and discharge respectively; EQ: 95% LOA −1173 to 877 kcal/day at discharge) (Supplementary figure 3a, 3b, and 3d respectively). A significant proportional bias was observed for energy intake versus target at the inclusion in the EQ group (β: 0.771, p = 0.003) (Supplementary figure 3c).

Within the IC group, the protein intake was comparable to the protein target both at inclusion [MD (95% CI) −0.1 (−0.5; 0.3) g/kg/day] and discharge [MD (95% CI) −0.1 (−0.3; 0.4) g/kg/day]. In the EQ group, the protein intake was significantly lower than the target both at inclusion and discharge [MD (95% CI) −0.2 (−0.3; 0.0) g/kg/day, p = 0.048 and −0.3 (−0.5; −0.1) g/kg/day, p = 0.004, respectively]. Bland-Altman plots for protein intake versus target showed wide LOA both at inclusion and discharge in both IC and EQ groups at individual level (IC: 95% LOA −81 to 67 and −97 to 79; EQ: 95% LOA −70 to 44 and −64 to 34 g/day at inclusion and discharge respectively) (Supplementary figure 4a, 4b, 4c and 4d respectively).

### Weight goal achievement

The absolute weight change was comparable between groups (median [IQR] IC: 0.2 [−1.3; 1.9], EQ: 0.0 [−1.9; 1.4] kg, MD for IC-EQ (95% CI) 0.4 (−0.9; 1.6) kg, p = 0.539). The goals to gain, maintain or lose weight were set by the dietitian in 9/22, 12/22, and 1/22 patient in the IC group and in 9/31, 22/31, and 0/31 patients in the EQ group respectively. Weight goals were successfully achieved by 15 out of 22 patients (68.2%) in the IC group and 16 out of the 31 patients (51.6%) in the EQ group. There was no between-group difference in the proportion of patients achieving weight goals (Table 3). The odds of weight goal achievement in the IC group compared to the EQ group was 2.01 (95% CI 0.64; 6.29).

**Table 3:** Weight change and weight goal achievements in indirect calorimetry and equation groups

	**Indirect calorimetry**	**Equation**	**p-value**
Weight change, kg, median [IQR]	0.2 [−1.3; 1.9]	0.0 [−1.9; 1.4]	0.539
Goal achievement			
Weight gain, n/N (%)	5/9 (55.6)	3/9 (33.3)	0.319
Weight maintenance, n/N (%)	9/12 (75.0)	13/22 (59.1)	0.249
Weight loss, n/N (%)	1/1 (100.0)	NA	NA
Total, n/N (%)	15/22 (68.2)	16/31 (51.6)	0.168


IQR: Interquartile range, NA: Not applicable, n/N: number of patients achieved weight goal/number of patients intended to achieve the weight goal

### Muscle mass, muscle strength, physical and functional performance

Table 4 presents the changes in muscle mass, physical and functional performance from admission to discharge in the IC and EQ groups. The CST score improved within the IC group and the gait speed improved within the EQ group. Total SPPB score, ADL, and IADL scores improved within both groups. No between group differences were found in the changes in all the secondary outcomes from admission to discharge.

**Table 4:** Changes in muscle mass, muscle strength, physical and functional performance in indirect calorimetry and equation groups

**Outcome measures**	**Indirect calorimetry group**					**Equation group**					**Between groups**	
	**Admission**		**Discharge**		**Mean change [95% CI]**	**Admission**		**Discharge**		**Mean change [95% CI]**	**Mean difference [95% CI]**	**p-value**
	**n**	**Value**	**n**	**Value**		**n**	**Value**	**n**	**Value**			
ALM, kg	19	18.5 [15.0–21.6]	18	20.1 [17.3–24.9]	0.5 [−0.9–1.8] ǂ	20	21.0 [18.2–24.0]	18	20.0 [19.2–24.5]	1.5 [−2.2–5.2]	−1.0 [−4.2; 2.2]	0.755
ALMI, kg/m^2^	19	6.5 [6.0–8.0]	18	7.1 [6.5–7.9]	0.2 [−0.3–0.6] ǂ	20	7.7 [6.7–8.9]	18	7.4 [6.7–8.3]	0.7 [−0.7–2.0]	−0.5 [−1.6; 0.7]	0.787
Handgrip strength, kg	21	20.5 [15.0–25.0]	22	19.0 [15.0–26.0]	0.1 [−2.3–2.4]	27	17.0 [10.5–22.0]	24	18.0 [13.5–24.0]	1.4 [−1.5–4.3]	−1.3 [−4.9; 2.2]	0.605
CST, score	22	0 [0; 1]	21	1 [0; 2]	0.5 [0.0; 1.0]*	28	0 [0; 0]	26	0 [0; 1]	0.2 [−0.2; 0.5]	−0.3 [−0.9; 0.2]	0.261
Gait speed, m/s	22	0.46 [0.00; 0.64]	21	0.61 [0.46–0.76]	0.19 [0.08–0.32] ǂ	28	0.00 [0.00–0.38]	25	0.49 [0.29–0.71]	0.31 [0.18–0.46]*	−0.06 [−0.33; 0.19]	0.437
SPPB, score	22	3 [0–6]	21	6 [3–8]	2[1–3]*	28	0 [0–3]	24	4 [2–7]	2 [1–4]*	1 [−1; 4]	0.675
Katz-ADL, score	22	2 [1–3]	20	5 [4–6]	3 [2–3]*	31	1 [1–2]	30	5 [2–5]	2 [2–3]*	0 [−1; 2]	0.570
Lawton-IADL, score	22	1 [0–2]	20	5 [2–6]	2[1–3]*	31	1 [1–2]	30	4 [2–5]	2[1–3]*	1 [−1; 2]	0.256


ALM: Appendicular lean mass; ALMI: Appendicular lean mass index; SPPB: Short physical performance battery; ADL: Activities of daily living; IADL: Instrumental ADL; Data are presented as median [IQR] unless indicated otherwise. Within-group differences (p<0.05 marked by *, p<0.10 marked by ǂ)

## Discussion

In this cohort of geriatric rehabilitation inpatients, nutritional interventions informed by indirect calorimetry compared to predictive equations did not lead to greater improvements in weight goal achievement or improvement in muscle mass, muscle strength, physical and functional performance. Despite the significant differences in measured RMR versus estimated RMR and the between group difference in energy targets, the absence of between group differences in outcomes can possibly be due the lack of agreement between energy intake and energy targets in patients within both groups.

RMR and energy targets were significantly overestimated by the equations, which aligns with the findings of a systematic review that none of the commonly used equations accurately predicted RMR in healthy older adults and with a prevalent tendency for overestimation (37). This overestimation may be due to factors known to reduce RMR in older adults such as decrease in fat-free mass and decrease in metabolic activity (11) that are not fully incorporated in most equations as the they are originally developed for adults. While RMR decreases with aging, it can also fluctuate due to different diseases and their stages (38), clinical conditions impacting body weight and body composition, and low physical activity amongst others (39). Such individual variations are taken into account when measuring RMR to subsequently determine the energy targets and therefore could help prevent over and underfeeding. However, our findings showed no greater benefits in weight goal achievement or improving muscle mass by using energy targets informed by measured RMR in geriatric rehabilitation inpatients. Measurement of RMR at the dietitian's initial assessment could facilitate accurate prescription of individualised energy targets in this population, but more evidence is needed to confirm the beneficial effects on clinical outcomes by utilising measured RMR in nutrition interventions in geriatric inpatients.

Energy targets in the IC group were lower than the EQ group, but there were no between group significant differences in the energy and protein intake. Within both groups, the energy and protein targets and the intake showed poor agreement, with many patients in negative energy and protein balance, which is in line with other studies in geriatric patients (40, 41). High prevalence of poor appetite, delirium, infection, cancer and assistance required for feeding in older patients have shown to be associated with inadequate energy intake (42). This could partly explain why patients were unable to meet energy targets in NEED. In addition, the energy served to patients in both groups were higher than the energy targets. Therefore, the poor agreement between energy targets and intake and energy served in both groups may explain the absence of between group differences in outcomes. Similar to our findings, enteral nutritional interventions guided by measured RMR compared to equations have also failed to show greater improvements in clinical outcomes such as the duration of mechanical ventilation and healing pressure sores in a sample of 27 adult patients admitted to a long term acute care hospital in which the energy delivery was also lower than the targets in both groups (43).

Meeting individualised energy and protein targets remained a challenge in geriatric rehabilitation inpatients despite receiving individualised nutritional interventions such as food fortification, oral nutrition supplementation and protein and energy enriched snacks that are shown to improve energy and protein intake in older inpatients (44, 45). Further strategies to optimise energy and protein intake may be beneficial, such as more intensive dietitian interventions including frequent intake monitoring and review of therapeutic diets, improvements in the food service systems, improved dining environments, and assistance during meal selection and mealtimes. Additionally, our findings emphasize the necessity of trying to adapt hospital food service systems to more closely deliver food to meet patient's individual energy and protein targets.

### Strengths and limitations

To our knowledge, NEED is the first study to investigate if nutritional interventions informed by indirect calorimetry compared to predictive equations lead to better clinical outcomes in geriatric rehabilitation inpatients. The use of the CGA with validated assessment methods appropriate for older patients and the nutritional intervention performed by dietitians are notable strengths of the study.

The quasi-experimental design enabled us testing the hypothesis in a real-life clinical setting. Despite the nutritional interventions are informed by IC measurement or equations, their delivery could have been influenced by the lack of control over energy served and energy intake, therewith the findings cannot support definitive conclusions. Moreover, the IC measurements were not performed in a fasted state due to practical limitations in the clinical setting and thus the RMR may be influenced by the thermic effect of food. However, the macronutrient composition of the meal consumed before the RMR measurement was comparable between the groups (supplementary table 1). The small sample size further limited by early discontinuation of patient recruitment and the between group differences in a few baseline characteristics and the length of stay may have limited identifying potential intervention effects. Despite no statistical significance, the higher odds of achieving weight goals by patients in the IC group compared to EQ group highlights the need to test the hypothesis in a large randomised controlled trial. The findings of this study will help design such trial.

## Conclusion

In this cohort of geriatric rehabilitation inpatients, nutritional interventions informed by indirect calorimetry compared to predictive equations did not lead to greater improvements in achieving weight goals or muscle mass, muscle strength, physical and functional performance. Identifying potential intervention effect was limited due to the lack of control over patients' energy intake, energy served and energy targets. Further, adequately powered randomised control trials are required to determine if using measured RMR compared to estimated RMR to guide nutritional interventions lead to better clinical outcomes, with a focus on matching the energy served and intake to targets.

## Electronic Supplementary Material

Supplementary material, approximately 179 KB.

## References

[1] Clark AB, Reijnierse EM, Lim WK, Maier AB (2020). Prevalence of malnutrition comparing the GLIM criteria, ESPEN definition and MST malnutrition risk in geriatric rehabilitation patients: RESORT. Clin Nutr.

[2] Wojzischke J, van Wijngaarden J, van den Berg C, Cetinyurek-Yavuz A, Diekmann R, Luiking YC, Bauer JM (2020). Nutritional status and functionality in geriatric rehabilitation patients: a systematic review and meta-analysis. Eur Geriatr Med.

[3] Marshall S, Bauer J, Isenring E (2014). The consequences of malnutrition following discharge from rehabilitation to the community: a systematic review of current evidence in older adults. J Hum Nutr Diet.

[4] van Wijngaarden JP, Wojzischke J, van den Berg C, Cetinyurek-Yavuz A, Diekmann R, Luiking YC, Bauer JM (2020). Effects of Nutritional Interventions on Nutritional and Functional Outcomes in Geriatric Rehabilitation Patients: A Systematic Review and Meta-Analysis. JAMDA.

[5] Holdoway A, Page F, Bauer J, Dervan N, Maier AB (2022). Individualised Nutritional Care for Disease-Related Malnutrition: Improving Outcomes by Focusing on What Matters to Patients. Nutrients.

[6] Volkert D, Beck AM, Cederholm T, Cruz-Jentoft A, Goisser S, Hooper L, Kiesswetter E, Maggio M, Raynaud-Simon A, Sieber CC, Sobotka L, van Asselt D, Wirth R, Bischoff SC (2019). ESPEN guideline on clinical nutrition and hydration in geriatrics. Clin Nutr.

[7] Gaddey HL, Holder KK (2021). Unintentional Weight Loss in Older Adults. Am Fam Physician.

[8] Hill JO, Wyatt HR, Peters JC (2013). The Importance of Energy Balance. Eur Endocrinol.

[9] Manini TM (2010). Energy expenditure and aging. Ageing Res Rev.

[10] Oshima T, Berger MM, De Waele E, Guttormsen AB, Heidegger CP, Hiesmayr M, Singer P, Wernerman J, Pichard C (2017). Indirect calorimetry in nutritional therapy. A position paper by the ICALIC study group. Clin Nutr.

[11] Rattanachaiwong S, Singer P (2019). Indirect calorimetry as point of care testing. Clin Nutr.

[12] Neelemaat F, van Bokhorst-de van der Schueren MA, Thijs A, Seidell JC, Weijs PJ (2012). Resting energy expenditure in malnourished older patients at hospital admission and three months after discharge: predictive equations versus measurements. Clin Nutr.

[13] Segadilha N, Rocha EEM, Tanaka LMS, Gomes KLP, Espinoza REA, Peres WAF (2017). Energy Expenditure in Critically Ill Elderly Patients: Indirect Calorimetry vs Predictive Equations. JPEN.

[14] World Medical Association Declaration of Helsinki (2013). Ethical Principles for Medical Research Involving Human Subjects. JAMA.

[15] Hooper R. Justify sample size for a feasibility study. RDS London. (Accessed 2 October, 2022, at https://www.rds-london.nihr.ac.uk/resources/justify-sample-size-for-a-feasibility-study/.)

[16] Rockwood K, Song X, MacKnight C, Bergman H, Hogan DB, McDowell I, Mitnitski A (2005). A global clinical measure of fitness and frailty in elderly people. CMAJ.

[17] Charlson M, Szatrowski TP, Peterson J, Gold J (1994). Validation of a combined comorbidity index. J Clin Epidemiol.

[18] Folstein MF, Folstein SE, McHugh PR (1975). “Mini-mental state”. A practical method for grading the cognitive state of patients for the clinician. J Psychiatr Res.

[19] Nasreddine ZS, Phillips NA, Bédirian V, Charbonneau S, Whitehead V, Collin I, Cummings JL, Chertkow H (2005). The Montreal Cognitive Assessment, MoCA: a brief screening tool for mild cognitive impairment. J Am Geriatr Soc.

[20] Storey JE, Rowland JT, Basic D, Conforti DA, Dickson HG (2004). The Rowland Universal Dementia Assessment Scale (RUDAS): a multicultural cognitive assessment scale. Int Psychogeriatr.

[21] Inouye SK, van Dyck CH, Alessi CA, Balkin S, Siegal AP, Horwitz RI (1990). Clarifying confusion: the confusion assessment method. A new method for detection of delirium. Ann Intern Med.

[22] Chumlea WC, Roche AF, Steinbaugh ML (1985). Estimating stature from knee height for persons 60 to 90 years of age. J Am Geriatr Soc.

[23] Ferguson M, Capra S, Bauer J, Banks M (1999). Development of a valid and reliable malnutrition screening tool for adult acute hospital patients. Nutrition.

[24] Vellas B, Guigoz Y, Garry PJ, Nourhashemi F, Bennahum D, Lauque S, Albarede JL (1999). The Mini Nutritional Assessment (MNA) and its use in grading the nutritional state of elderly patients. Nutrition.

[25] Weir JBV (1949). New methods for calculating metabolic rate with special reference to protein metabolism. Physiol J.

[26] Lupinsky L, Singer P, Theilla M, Grinev M, Hirsh R, Lev S, Kagan I, Attal-Singer J (2015). Comparison between two metabolic monitors in the measurement of resting energy expenditure and oxygen consumption in diabetic and non-diabetic ambulatory and hospitalized patients. Nutrition.

[27] Yeung SSY, Trappenburg MC, Meskers CGM, Maier AB, Reijnierse EM (2020). The use of a portable metabolic monitoring device for measuring RMR in healthy adults. Br J Nutr.

[28] Schofield WN (1985). Predicting basal metabolic rate, new standards and review of previous work. Hum Nutr Clin Nutr.

[29] CBORD Room Service Choice. (Accessed 2 October, 2022, at https://www.cbord.com/industries/healthcare/)

[30] Farris G, Mattison M, McKean SC, Ross JJ, Dressler DD, Scheurer DB (2017). Principles and Practice of Hospital Medicine, 2e.

[31] Verstraeten LMG, van Wijngaarden JP, Kim DY, Meskers CGM, Maier AB (2023). Feasibility of bioelectrical impedance analysis in routine clinical care to assess body composition in geriatric rehabilitation inpatients: RESORT. Aging Clin Exp Res.

[32] Reijnierse EM, de Jong N, Trappenburg MC, Blauw GJ, Butler-Browne G, Gapeyeva H, Hogrel JY, McPhee JS, Narici MV, Sipila S, Stenroth L, van Lummel RC, Pijnappels M, Meskers CGM, Maier AB (2017). Assessment of maximal handgrip strength: how many attempts are needed?. J Cachexia Sarcopenia Muscle.

[33] Guralnik JM, Simonsick EM, Ferrucci L, Glynn RJ, Berkman LF, Blazer DG, Scherr PA, Wallace RB (1994). A short physical performance battery assessing lower extremity function: association with self-reported disability and prediction of mortality and nursing home admission. J Gerontol.

[34] Katz S, Ford AB, Moskowitz RW, Jackson BA, Jaffe MW (1963). Studies of Illness in the Aged. The Index of Adl: A Standardized Measure of Biological and Psychosocial Function. JAMA.

[35] Lawton MP, Brody EM (1969). Assessment of older people: self-maintaining and instrumental activities of daily living. Gerontologist.

[36] Martin Bland J, Altman D (1986). Statistical methods for assessing agreement between two methods of clinical measurement. The Lancet.

[37] Cioffi I, Marra M, Pasanisi F, Scalfi L (2020). Prediction of resting energy expenditure in healthy older adults: A systematic review. Clin Nutr.

[38] Elia M, Ritz P, Stubbs RJ (2000). Total energy expenditure in the elderly. Eur J Clin Nutr.

[39] Achamrah N, Delsoglio M, De Waele E, Berger MM, Pichard C (2021). Indirect calorimetry: The 6 main issues. Clin Nutr.

[40] Weijzen MEG, Kouw IWK, Verschuren AAJ, Muyters R, Geurts JA, Emans PJ, Geerlings P, Verdijk LB, van Loon LJC (2019). Protein Intake Falls below 0.6 g*kg-1*d-1 in Healthy, Older Patients Admitted for Elective Hip or Knee Arthroplasty. J Nutr Health Aging.

[41] Weijzen MEG, Kouw IWK, Geerlings P, Verdijk LB, van Loon LJC (2020). During Hospitalization, Older Patients at Risk for Malnutrition Consume <0.65 Grams of Protein per Kilogram Body Weight per Day. Nutr Clin Pract.

[42] Mudge AM, Ross LJ, Young AM, Isenring EA, Banks MD (2011). Helping understand nutritional gaps in the elderly (HUNGER): a prospective study of patient factors associated with inadequate nutritional intake in older medical inpatients. Clin Nutr.

[43] Landes S, McClave SA, Frazier TH, Lowen CC, Hurt RT (2016). Indirect Calorimetry: Is it Required to Maximize Patient Outcome from Nutrition Therapy?. Curr Nutr Rep.

[44] Mills SR, Wilcox CR, Ibrahim K, Roberts HC (2018). Can fortified foods and snacks increase the energy and protein intake of hospitalised older patients? A systematic review. J Hum Nutr Diet.

[45] Collins J, Porter J, Truby H, Huggins CE (2017). A foodservice approach to enhance energy intake of elderly subacute patients: a pilot study to assess impact on patient outcomes and cost. Age Ageing.

