# Supplementary material for: Nutrition Intervention Informed by Indirect Calorimetry Compared to Predictive Equations to Achieve Weight Goals in Geriatric Rehabilitation Inpatients: The NEED Study
Source: J Nutr Health Aging. 2023 Sep 23;27(10):833–41. doi: 10.1007/s12603-023-1970-5 (PMC12930051; doi:10.1007/s12603-023-1970-5)
Supplement: Supplementary file 1 — Supplementary material, approximately 179 KB. [file mmc1.docx]

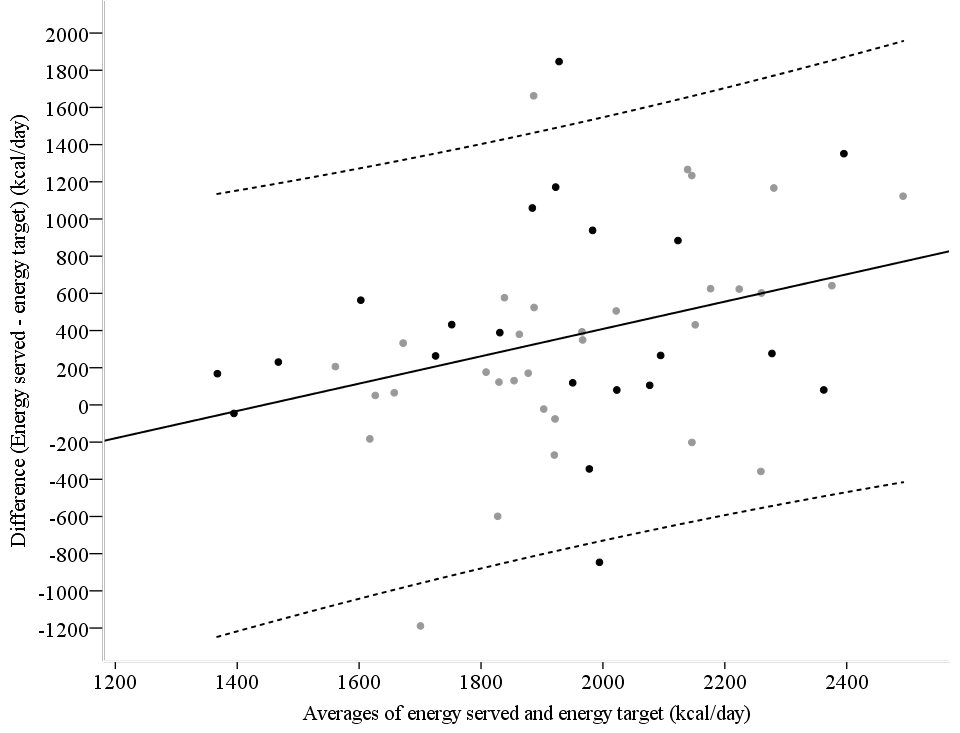


**Supplementary figure 2.** Bland-Altman plot of the difference versus averages of measured and estimated resting metabolic rate (RMR) in patients.

Black dots represent the indirect calorimetry group (n=22) and grey dots represent the equation group (n=31). The solid middle lines represent the mean differences in measured and estimated resting RMR, while the dashed lines at its sides represent the upper and lower 95% limit of agreement (mean difference±1.96 SD). No proportional bias was observed between the measured versus estimated RMR.


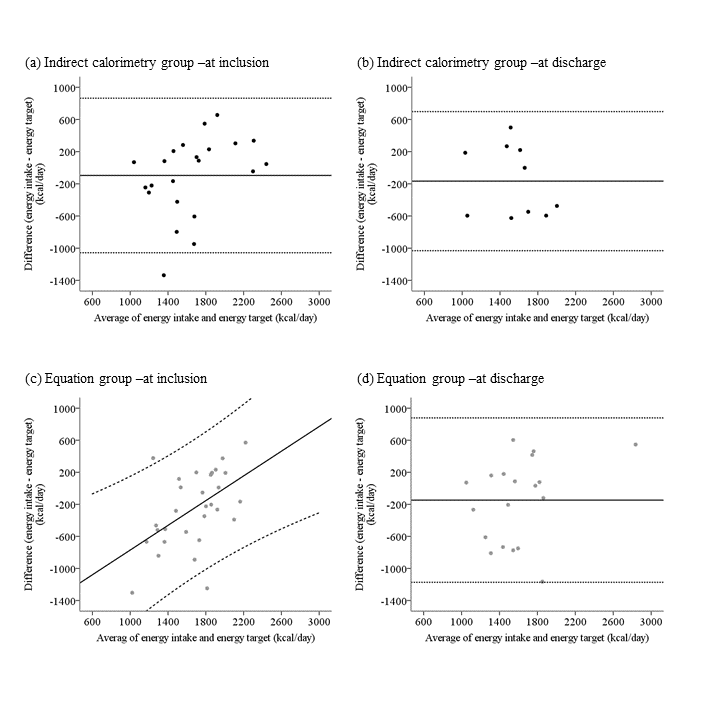


**Supplementary figure 3.** Bland-Altman plots of energy intake and energy target at inclusion and discharge among patients in the indirect calorimetry group (n=22 and n=10) (a, b) and the equation group (n=31 and n=19) (c, d). The solid middle lines represent the mean differences in energy intake and energy target, while the dashed lines at its sides represent the upper and lower 95% limit of agreement (mean difference±1.96 SD) (a, b, d). Proportional bias for energy intake versus target was observed at the inclusion in the equation group; the solid lines represent the expected differences in energy intake and target, while the dashed lines represent the regression-based upper and lower 95% limit of agreement (expected difference±1.96 SD) (c).


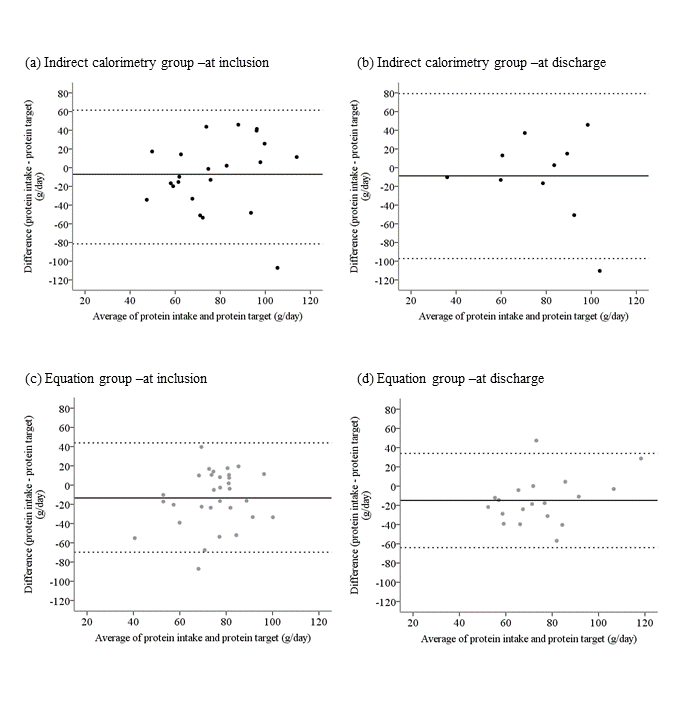


**Supplementary figure 4**. Bland-Altman plots of protein intake and protein target at inclusion and discharge among patients in the indirect calorimetry group (n=22 and n=10) (a, b) and the equation group (n=31 and n=19) (c, d). The solid middle lines represent the mean differences in protein intake and protein target, while the dashed lines at its sides represent the upper and lower 95% limit of agreement (mean difference±1.96 SD).

**Supplementary Table 1. Energy intake and the macronutrient composition of the meal prior to RMR measurement in the indirect calorimetry and equation groups**

| Intake at breakfast | Indirect calorimetry group | Equation group |
| --- | --- | --- |
| Energy (kcal) | 392.3 [219.2; 535.1] | 419.1 [276.0; 542.2] |
| % contribution by protein | 16.7 [12.2; 19.6] | 16.9 [12.4; 20.2] |
| % contribution by fat | 23.4 [16.5; 29.6] | 26.3 [20.2; 35.0] |
| % contribution by carbohydrate | 59.0 [50.6; 62.2] | 55.1 [46.1; 60.8] |
